# Dysregulation at multiple points of the kynurenine pathway is a ubiquitous feature of renal cancer: implications for tumour immune evasion

**DOI:** 10.1038/s41416-020-0874-y

**Published:** 2020-05-11

**Authors:** Nick Hornigold, Karen R. Dunn, Rachel A. Craven, Alexandre Zougman, Sebastian Trainor, Rebecca Shreeve, Joanne Brown, Helen Sewell, Michael Shires, Margaret Knowles, Tsutomu Fukuwatari, Eamonn R. Maher, Julie Burns, Selina Bhattarai, Mini Menon, Alvis Brazma, Ghislaine Scelo, Lara Feulner, Yasser Riazalhosseini, Mark Lathrop, Adrian Harris, Peter J. Selby, Rosamonde E. Banks, Naveen S. Vasudev

**Affiliations:** 1 Clinical and Biomedical Proteomics Group, University of Leeds, St. James’s University Hospital, Beckett Street, Leeds, LS9 7TF UK; 2 Leeds Institute of Medical Research at St James’s, University of Leeds, St. James’s University Hospital, Beckett Street, Leeds, LS9 7TF UK; 3 Molecular Genetics Group, University of Leeds, St. James’s University Hospital, Beckett Street, Leeds, LS9 7TF UK; 4 Department of Nutrition, The University of Shiga Prefecture, 2500 Hassaka, Hikone, 5228533 Japan; 5 Department of Medical Genetics, University of Cambridge and NIHR Cambridge Biomedical Research Centre, and Cancer Research UK Cambridge Centre, Cambridge Biomedical Campus, Cambridge, CB2 0QQ UK; 6 Department of Pathology, St James’s University Hospital, Beckett Street, Leeds, LS9 7TF UK; 7 European Molecular Biology Laboratory, European Bioinformatics Institute, EMBL-EBI, Wellcome Trust Genome Campus, Hinxton, CB10 1SD UK; 8 International Agency for Research on Cancer (IARC), Genetic Epidemiology Group, 150 cours Albert Thomas, 69372 Lyon, France; 9 McGill University and Genome Quebec Innovation Centre, 740 Doctor Penfield Avenue, Montreal, QC H3A 0G1 Canada; 10 Cancer Research UK Clinical Centre, Weatherall Institute of Molecular Medicine, John Radcliffe Hospital, Headington, Oxford OX3 9DS UK

**Keywords:** Renal cell carcinoma, Cancer metabolism

## Abstract

**Background:**

Indoleamine 2,3-dioxygenase (IDO), the first step in the kynurenine pathway (KP), is upregulated in some cancers and represents an attractive therapeutic target given its role in tumour immune evasion. However, the recent failure of an IDO inhibitor in a late phase trial raises questions about this strategy.

**Methods:**

Matched renal cell carcinoma (RCC) and normal kidney tissues were subject to proteomic profiling. Tissue immunohistochemistry and gene expression data were used to validate findings. Phenotypic effects of loss/gain of expression were examined in vitro.

**Results:**

Quinolate phosphoribosyltransferase (QPRT), the final and rate-limiting enzyme in the KP, was identified as being downregulated in RCC. Loss of QPRT expression led to increased potential for anchorage-independent growth. Gene expression, mass spectrometry (clear cell and chromophobe RCC) and tissue immunohistochemistry (clear cell, papillary and chromophobe), confirmed loss or decreased expression of QPRT and showed downregulation of other KP enzymes, including kynurenine 3-monoxygenase (KMO) and 3-hydroxyanthranilate-3,4-dioxygenase (HAAO), with a concomitant maintenance or upregulation of nicotinamide phosphoribosyltransferase (NAMPT), the key enzyme in the NAD+ salvage pathway.

**Conclusions:**

Widespread dysregulation of the KP is common in RCC and is likely to contribute to tumour immune evasion, carrying implications for effective therapeutic targeting of this critical pathway

## Background

Renal cancer is one of the ten most common adult cancers, accounting for over 100,000 deaths worldwide each year.^1^ It is also a cancer with one of the highest projected increases in incidence over the next two decades.^2^ Almost 90% of these cancers arise within the renal parenchyma and are termed renal cell carcinomas (RCCs). The most common (75%) histological subtype is clear cell RCC (conventional) (ccRCC), which are characterised by loss of the *VHL* tumour-suppressor gene, followed by papillary (10–15%) and chromophobe (5%) RCC. Each is considered to arise from distinct parts of the human nephron, are genetically distinct^3^ and vary considerably in their clinical behaviour.

Treatment options for patients with RCC have burgeoned in recent years, but despite this, it is a cancer that remains incurable for most patients with advanced disease. As in a number of other tumour types, immunotherapy, in the form of checkpoint inhibitors (CPIs), has come to the forefront of patient treatment, both in the first- and second-line settings. However, while some patients are observed to have deep and durable responses to these agents, many patients fail to respond.^4^ It appears therefore that, alone, CPIs are not always sufficient to overcome immune evasion and immune tolerance by tumours. Hence, increasing attention is being focussed on combining these drugs with other immune-modifying targeted agents.^5^

Alterations in cellular metabolism are a hallmark of cancer,^6^ most notably perhaps the ‘Warburg effect’, that describes the increased rate of glycolysis with reduced oxidative phosphorylation characteristic of tumours.^7^ We were among the first to demonstrate this comprehensively in renal cancers, using a proteomic approach, showing an upregulation in the majority of proteins in the glycolytic pathway and a parallel downregulation of mitochondrial enzymes in comparison to normal renal tissues,^8^ highlighting novel opportunities for therapeutic targeting.^9^

More recently, alteration in the metabolism of the essential amino acid tryptophan in cancer, through the kynurenine pathway, has come to wide attention as a mechanism by which tumours may escape immune control and promote disease progression. The enzymes indoleamine 2,3-dioxygenase (IDO1, IDO2) and tryptophan 2,3-dioxygenase (TDO) initiate the first steps in the kynurenine pathway, converting tryptophan to kynurenine, with the TDO-dependent pathway in the liver normally accounting for the majority of tryptophan metabolism and IDO-mediated metabolism predominantly occurring secondary to inflammation and cytokine-induced upregulation.^10^ In normal physiology, IDO plays an important role in tolerance to non-self-antigens, for example foetal antigens, where such immune non-responsiveness may be important.^11,12^ Upregulation of IDO leads to tryptophan depletion and kynurenine accumulation, which appear to work in concert to mediate immunosuppression, via T cell anergy and apoptosis and suppressed T cell differentiation.^13^ The harnessing of this phenomenon by tumours has led to the development of inhibitors of IDO1 that have progressed to clinical trials in combination with CPI. Despite much promise, initial results have, however, been disappointing and the future of these agents currently remains uncertain.^14,15^ This may be because of patient selection and lack of suitable profiling of immuno-regulating metabolism, emphasising the need for a deeper understanding of these pathways.

Here, using a proteomic-based approach, we show that the kynurenine pathway is more broadly disrupted than has been previously considered, extending beyond IDO1, that this is a common event in RCC and is not just restricted to the clear cell histological subtype and suggests possible redundancy in the pathway within the tumour setting. Our findings are of significance in terms of highlighting various aspects of this pathway for potential therapeutic targeting, patient stratification and may have implications for other cancers.

## Methods

### Reagents

Reagents were purchased as follows: general chemicals (Sigma, Poole, UK and VWR, Poole, UK), goat serum and human serum albumin, mouse monoclonal anti-β-actin antibody clone AC15 (Sigma), Hybond™C super NC membrane, Pharmalyte pH 3–10, IPG strips, dry strip cover fluid, bromophenol blue and PlusOne Silver Stain (GE Healthcare,Little Chalfont, UK), CHAPS (Calbiochem, San Diego, USA), LMP agarose, Minimum Essential Medium (MEM)-Alpha medium, L-glutamine, trypsin with EDTA, G418, HBSS and Antibody Diluent (Invitrogen Life Technologies, Paisley, UK), foetal calf serum (FCS; Harlan-Seralab, Sussex, UK); PBS (Oxoid, Basingstoke, UK), acrylamide (National Diagnostics, Hull, UK), OWL Silver Stain (OWL Separation Systems, Portsmouth, USA), trypsin sequencing grade (Promega, Southampton, UK), ACN (Rathburn, Walkerburn, UK), Complete™ mini protease inhibitor cocktail tablets (Roche, Lewes, UK), Envision™1 systems (Dako, Ely, UK), SuperSignal® West Dura Extended Duration Substrate (Pierce, Tattenhall, UK), Access Revelation solution (Menarini Diagnostics, Berkshire, UK), Bloxall, Impress Rabbit horseradish peroxidase (HRP)‐conjugated secondary antibodies, Impact DAB and horse serum (Vector Laboratories, Peterborough, UK), BCA protein assay (Thermo Scientific, Warrington, UK) mouse monoclonal anti-VHL clone Ig32 (BD Biosciences, Wokingham, UK), affinity-purified rabbit antibodies to kynurenine pathway components KMO (cat. nos. HPA056942 and HPA031115), KYNU (cat. no.HPA031686), NAMPT (cat. no. HPA047776) and IDO1 (cat. no. HPA023072) (Atlas Antibodies, Sweden), oligonucleotides (Eurogentec).

### Established cell line VHL transfectants

Cell line pairs generated from the *VHL*-defective human RCC cell lines UMRC2, RCC4 and 786-0 by stable transfection with either empty vector or a wild-type (WT) *VHL* expression construct have been described previously.^16–18^ 786-0, stably transfected with full-length *VHL* (786+VHL) or control vector (786+pRC) were obtained as gifts from W.G. Kaelin.^16^ Cells were maintained in MEM-α medium supplemented with 10% v/v FCS and 1% v/v L-glutamine, as previously described.^19^ All cell lines were screened for mycoplasma contamination.

### Quinolate phosphoribosyltransferase (QPRT) expression and QPRT knockdown stable transfectants

*QPRT* gene expression constructs containing empty vector (pFB-HYG) or QPRT (pFB-HYG-QPRT) were created. The QPRT insert was made by PCR of human cDNA (forward primer: *GTCAGTCGACCACCATGGACGCTGAAGGCC* and reverse primer *GACTCGAGCTAGTGGATTTTGGGCACTGGAGC*) followed by digestion with Sal1 and Xho1 and ligation into the multiple cloning site of pFB. Correct sequence was confirmed by sequencing. These constructs were amplified in XL1 Blue competent *Escherichia coli* (Stratagene), then introduced into 293-PhoenixA cells using SiPORT transfection agent (Ambion). Supernatant containing viral particles was harvested on days 3 and 4, and polybrene was added to 8 μg/ml. Four ml of medium was used to infect each T75 flask of a *VHL*-defective 786-0 cell line lacking endogenous VHL followed by selection with hygromycin (0.5 mg/ml) and were designated 786+pFB and 786+QPRT, respectively. Cells were maintained in MEM-α medium supplemented with 10% FCS, 1% v/v L-glutamine, G418 (1 mg/ml) and hygromycin selection (0.5 mg/ml).

*QPRT* short hairpin RNA (shRNA) constructs containing a non-specific shRNA (pRetroSuper-shRNA-scramble) or shRNA targeting *QPRT* (pRetroSuper-shRNA-QPRT) were created using the following oligonucleotides (capitals indicate nucleotides corresponding to QPRT sequence or control, lower case indicates loop and linker sequences):

shQPRT:

Forward *gatccccGCCCTTGATTTCTCCCTCAttcaagagaTGAGGGAGAAATCAAGGGCtttttggaaa*

Reverse *agcttttccaaaaaGCCCTTGATTTCTCCCTCAtctcttgaaTGAGGGAGAAATCAAGGGCggg*

Scramble:

Forward

*gatccccCTTCAGCCGTTACGCTCGGttcaagagaCCGAGCGTAACGGCTGAAGtttttggaaa*

Reverse

*agcttttccaaaaaCTTCAGCCGTTACGCTCGGtctcttgaaCCGAGCGTAACGGCTGAAGggg*

Oligonucleotide pairs were annealed by heating to 100 °C for 2 min and cooling slowly to room temperature and ligated into HindIII/BglII-digested pRetroSuper-puro (a gift from Darren Tomlinson). Constructs were amplified in XL1 Blue competent *E. coli* (Stratagene), then introduced into 293-PhoenixA cells using SiPORT transfection agent (Ambion). Supernatant containing viral particles was harvested on days 3 and 4, and polybrene was added to 8 μg/ml. Four ml of the medium was used to infect each T75 flask of 786+VHL cells, followed by selection with puromycin (2 μg/ml). Cell lines were maintained in MEM-α medium supplemented with 10% FCS, 1% v/v L-glutamine, G418 (1 mg/ml) and puromycin (2 µg/ml).

### Two-dimensional (2D) gel electrophoresis

For global protein profiling of 786-0 cells +/−VHL, protein extracts (80 µg protein for analytical gels and 1 mg for preparative gels) were analysed by 2D polyacrylamide gel electrophoresis (2D-PAGE) over a pH range of 4–7 using a combined IPGPhor and Multiphor approach.^20^ Protein samples were loaded onto IPG strips by overnight in-gel rehydration and focussing carried out for a total of 65 kVh. Strips were equilibrated in running buffer, placed onto polyacrylamide gels (10% resolving gel with 4% stacking gel) and electrophoresed overnight (12.5 °C, 18 mA/gel). Gels were stained using OWL silver stain and scanned using a Personal Densitometer SI (GE Healthcare), and images from triplicate gels were analysed using the Melanie 3 software. Preparative gels were stained with PlusOne™ Silver Stain using a modified staining protocol,^21^ and selected spots were excised and digested with trypsin.^22^ Peptides were analysed by Nano-LC (Ultimate, LC Packings (Dionex), Camberley, UK) followed by automated data-dependent mass spectrometry (MS)/MS using a Q-TOF mass spectrometer (Micromass, Manchester, UK). Protein identities were determined by searching the NCBI database using MS-TAG or MS-Pattern (prospector.ucsf.edu).

### Renal tissue samples

Frozen renal tissue samples from 42 previously untreated patients who had undergone nephrectomy for sporadic ccRCC from December 2001 to December 2006 were obtained from the Leeds Multidisciplinary Research Tissue Bank (REC Ref 15/YH/0080). Tissue collection and processing was as previously described.^19^ Ten pairs of matched tumour/normal tissue were used for Western blot analysis. For the initial immunohistochemical (IHC) studies of QPRT, frozen tissue sections from 13 tumours and matched normal kidney samples were examined. Further examination of additional proteins and across other RCC subtypes was achieved through a tissue microarray (TMA), containing formalin-fixed paraffin-embedded (FFPE) tissue cores from a further 20 patients, reviewed and selected by an experienced pathologist, from 5 normal renal cortex, 5 normal renal medulla, 11 ccRCC, 6 papillary RCC and 3 chromophobe RCC samples, each arrayed in duplicate. In addition, previously generated liquid chromatography (LC)-MS/MS proteomic data sets analysing 13 matched tumour/normal pairs of ccRCC tissues and 7 matched pairs of chromophobe RCC tissues were also interrogated for the purposes of this study. This data set forms part of a larger proteogenomic study (manuscript in preparation). All included tumours were reviewed by an expert pathologist to confirm at least 70% viable tumour cells. Details of patients/tumours across the various substudies are presented in Supplementary Table 1.

### Immunocytochemistry, immunohistochemistry and western blotting for QPRT

Initial analysis of cell lines for QPRT was undertaken using affinity-purified rabbit antiserum to QPRT.^23^ For all other studies, custom rabbit antiserum to QPRT was raised (Eurogentec, Belgium) by immunising rabbits with peptides (CDLVLLDNFKPEELHP or CVAGTRKTTPGFRLVE).

Multi-well slides of 786-0+/−VHL cell lines were fixed in acetone for 2 min, air dried, washed briefly in TBS-T, endogenous peroxidase blocked using 0.6% v/v hydrogen peroxide in methanol for 5 min and washed again. After overnight incubation at 4 °C in rabbit antiserum to QPRT diluted 1:20,000 in TBS/0.1% w/v HSA with 0.1% w/v sodium azide, slides were washed in TBS-T and labelled using the rabbit EnVision+ detection system with DAB substrate according to the manufacturer’s instructions. Slides were counterstained with Mayer’s haematoxylin and mounted using DePeX mounting medium. Negative control sections were probed with an irrelevant antibody. Immunohistochemistry on sections of frozen tumour and normal tissue (5 µm OCT-embedded) was similarly performed.^22^

Western blotting of protein lysates of cell lines or matched tumour/normal tissue was performed as previously described^24^ with samples separated by 10% sodium dodecyl sulfate (SDS)-PAGE and transferred to Hybond-C Super NC membrane in Towbin’s buffer. After blocking with TBS-T/10% w/v dried skimmed milk, blots were probed with antibodies to QPRT (1:20,000), VHL (1 µg/mL) and β-actin (5 ng/ml; protein-loading control). After washing, blots were incubated with anti-rabbit or anti-mouse HRP-conjugated Envision+ reagent, then washed again and exposed to film. In all cases, western blots were normalised using densitometric scanning of parallel Coomassie blue-stained gels for total protein load, given the limitations of housekeeping genes.^25^ Additional blotting against beta actin was variably employed.

### Measurement of quinolinic acid (QUIN) in renal tissue samples

Sections from matched pairs of frozen tumour/normal tissue were cut into 1 M HCL and stored at −80 °C. QUIN levels were measured in Schwarz laboratory by gas chromatography MS, performed as previously described.^26^

### Effects of QPRT on cell proliferation

786+pFB and 786+QPRT cells in an exponential phase of growth were harvested and plated in 96-well plates (1 × 10^4^ cells/well) and cultured for 24, 48 and 72 h at 37 °C with 5% CO_2_/95% air. Viable cells were quantified using WST-1 reagent according to the manufacturer’s protocol, and absorbance was measured at 450 and 650 nm. Wells were seeded in triplicate, and three independent experiments were run.

### Effects of QPRT on anchorage-independent colony-formation assay

To investigate anchorage-independent growth of 786-0 cell lines stably transduced with constructs of interest, WT VHL (786+VHL)+shRNA scramble control, WT VHL (786+VHL)+shRNA QPRT, VHL negative+empty vector (786+pFB) and VHL negative+QPRT (786+QPRT) were cultured at 4 × 10^4^ cells/well as previously described.^27^ Viable colonies were stained with 8 mM p-iodonitrotetrazolium violet, and colonies with a diameter of >1 mm were then counted within 10 random fields of view using an eyepiece graticule (1 cm^2^ area, made up of 10 ×10 mm squares) on a bright field microscope. The mean number of colonies per 10 cm^2^ from four independent experiments was determined. Statistical significance was assessed by Student’s *T* test. The NIH3T3 cell lines containing either a control or H1047R vector were employed as negative and positive controls for the assay, respectively.

### Interrogation of LC-MS/MS RCC proteomic data sets for kynurenine pathway changes

As part of a large proteogenomic study of RCC (manuscript in preparation), LC-MS/MS proteomic data sets were created containing 13 matched tumour/normal pairs of ccRCC and 7 matched tumour/normal pairs of chromophobe RCC following expert pathological review of the selected blocks. We were able to interrogate this data for evidence of protein expression of enzymes from the kynurenine pathway. For each sample, 30-µm sections equivalent to 3 cm^2^ surface area of tissue were lysed in excess lysis solution (250 µl of 3% SDS in 50 mM Tris-HCl, pH 7.6), and DNA was sheared with brief sonication. Samples were then heated at 95 °C for 10 min, centrifuged at 13,000 × *g* for 8 min, supernatant removed, and protein concentration was measured by BCA assay. Dithiothreitol was added to samples at a final concentration of 30 mM, and samples were heated at 95 °C for 5 min. Seventy μg of protein was processed by the STrap protocol as previously described.^28^ Label-free MS and data analysis were conducted essentially as previously described^29^ but using an EASY-nLC 1000 UHPLC system connected to a capillary emitter column (75 μm inner diameter, packed with 3 μ Pursuit C_18_ media) hyphenated to an LTQ-Orbitrap Velos mass spectrometer (Thermo Fisher Scientific). Data were processed against the Uniprot human protein database using the Maxquant 1.3.0.5 software. Maximum false discovery rates were set to 0.01. PEP and *Q*-values calculate the probability of false identification for the proteins described in this study as being extremely low (*p* < 0.001).

### IHC analysis of the kynurenine pathway

TMA FFPE sections (4 μm) were mounted onto Plus Frost slides, and dewaxing and epitope recovery was carried out by heating in a Cookworks pressure cooker for 5 min on high followed by 25 min on low temperature in Access Revelation solution. Endogenous peroxidase activity was blocked in Bloxall and 2.5% v/v normal horse serum used as a protein block. Antibodies were optimally diluted in Antibody Diluent and detected using rabbit HRP‐conjugated secondary antibodies followed by Impact DAB substrate for 5 min at room temperature, then counterstained with Mayer’s haematoxylin for 30 s dehydrated, cleared in xylene and mounted in DPX.

## Results

### QPRT is downregulated in ccRCC

**Fig. 1:**
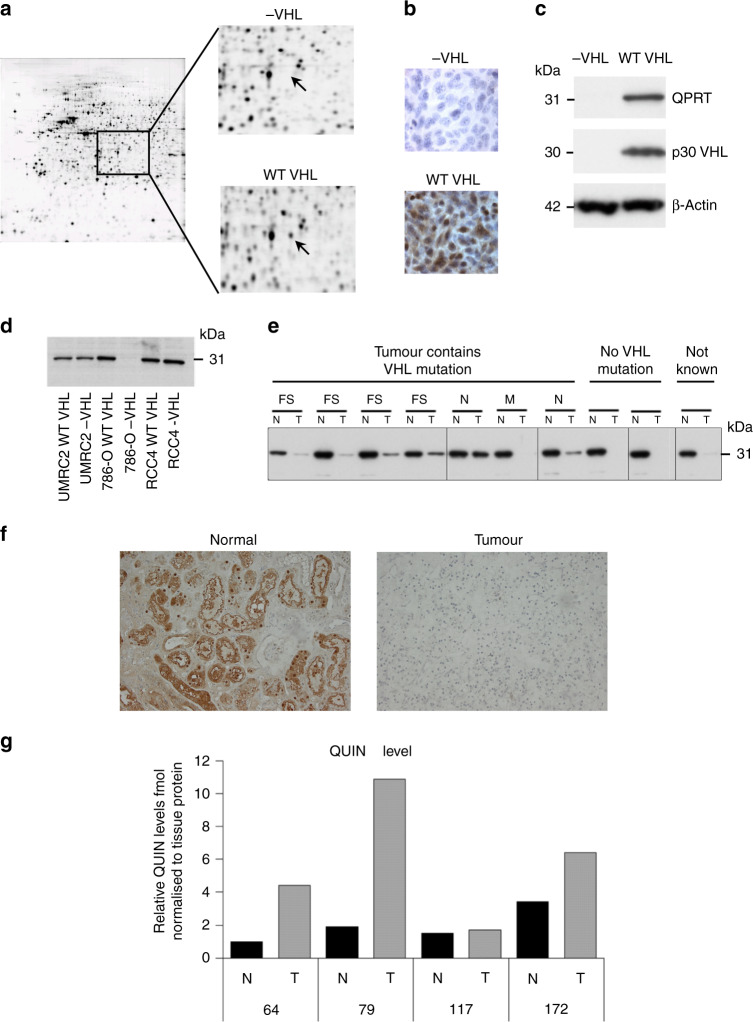
QPRT expression is lost in clear cell RCC (ccRCC). **a** 2D-PAGE comparing 786-0 cell lines, +/−wild-type *VHL*. The protein spot identified as QPRT is arrowed. **b** Immunocytochemistry of 786-0+/− cell lines for QPRT. **c**–**e** Western blotting for QPRT in **c** 786-0+/−VHL cell lines; **d** 786-0, RCC4 and UMRC2 cell lines +/−wild-type *VHL*; **e** Paired normal/ccRCC tumour tissue lysates. *VHL* mutation status is indicated (one tumour was of unknown *VHL* status: FS frame shift, N nonsense, M mis-sense). Promoter methylation status was analysed in one of the two tumours containing no *VHL* mutation and confirmed as negative **f** Immunohistochemistry for QPRT of a representative example of normal and ccRCC tissue (×40 magnification). **g** Quinolinic acid content of paired normal/ccRCC tumour tissue samples determined by mass spectrometry.

From our 2D-PAGE-based comparison of whole-cell lysates of 786-0−/+*VHL* cells, we identified QPRT as being undetectable in −*VHL* cells and expressed at relatively high levels following re-introduction of WT *VHL* (Fig. 1a and Supplementary Table 2). This was confirmed using immunocytochemistry and western blotting of the 786-0+/−*VHL* cell lines (Fig. 1b, c). However, this apparent VHL-dependent expression of QPRT was not observed in UMRC2 and RCC4+/−*VHL* cell line pairs (Fig. 1d), which all retained expression. Western blotting of frozen tissue lysates from ten ccRCC/normal kidney matched pairs with tumour *VHL* mutation status defined in all but one pair (Fig. 1e) showed significant loss or downregulation of QPRT expression in ccRCC tissues compared to their normal counterparts in nine cases, although no relationship with *VHL* mutation status or mutation type was apparent. The loss of QPRT in clear cell tumours was confirmed in 12/13 patients by IHC (Fig. 1f).

Taken together, these results indicate that loss of QPRT is a common event in ccRCC although regulation by VHL in vitro is cell line dependent. QPRT catalyses the conversion of QUIN, produced within the kynurenine pathway, to nicotinamide adenine dinucleotide (NAD+). The reaction catalysed by QPRT is a rate-limiting step in this pathway, therefore loss of QPRT may lead to an increase in the level of QUIN. In support of this hypothesis, we found increased levels of QUIN in ccRCC tissues relative to patient-matched normal kidney cortex (Fig. 1g).

### Knockdown of QPRT in 786-0 cells increases cellular anchorage-independent growth in vitro

**Fig. 2:**
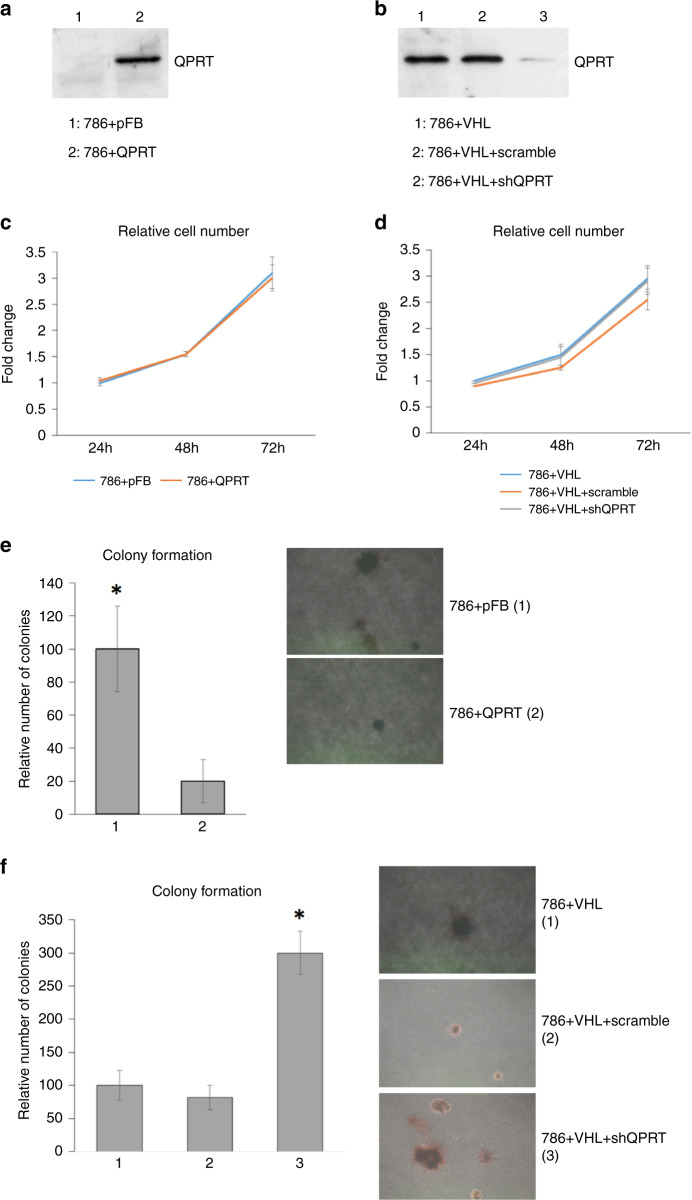
Loss of QPRT expression increases anchorage-independent growth. **a** Western blot showing QPRT expression in control and QPRT-transfected *VHL* negative 786-0 cell lines. **b** QPRT expression in control and anti-QPRT shRNA transfected, VHL-expressing 786-0 cell lines. A single band at the expected molecular weight was observed. **c**, **d** Relative cell number of 786-0 cell lines plus or minus QPRT, as measured using WST1 assay. **e**, **f** Impact of QPRT transfection or knockdown on relative colony number in soft agar colony-formation assay and representative images. Colony number per 10 cm^2^ are provided as a mean of the sum over four independent experiments and then standardised as a percentage to either 786+pRC or 786+VHL controls. Significant differences are indicated (asterisk (*); 786+QPRT versus 786+pFB, *p* = 0.017, 786+VHL+shQPRT versus either control, *p* = 0.001).

786-0 cells (VHL-negative) transfected with a QPRT expression construct (786+QPRT) showed stable overexpression of QPRT (Fig. 2a). Conversely, transfection of 786+VHL (VHL-expressing) cells with an shRNA construct targeting QPRT (786+VHL+shQPRT) cells significantly reduced QPRT protein expression (Fig. 2b). QPRT expression in cells transfected with empty vector was unaffected.

Using this model, no significant effect of QPRT loss/gain on cell proliferation was observed (Fig. 2c, d). However, in a soft agar colony-formation assay (Fig. 2e, f), whereas all 786-0 cells, irrespective of whether +/−VHL, formed small colonies (diameter of >1 mm), 786+QPRT cells showed significantly reduced colony formation as compared to 786+pRC controls (*p* = 0.017) (Fig. 2e) and 786+VHL+shQPRT cells showed a significantly (*p* = 0.001) higher frequency of colonies relative to both the 786+VHL+scramble and 786+VHL cells (Fig. 2f). Colony size was also affected by QPRT expression status. Among QPRT-positive cell lines, only 10% of colonies were estimated to exceed 3 mm in diameter, versus approximately 50% of colonies among QPRT-negative cells lines.

### Dysregulation of the kynurenine pathway occurs at multiple points and is common to both ccRCC and chromophobe RCC

**Fig. 3:**
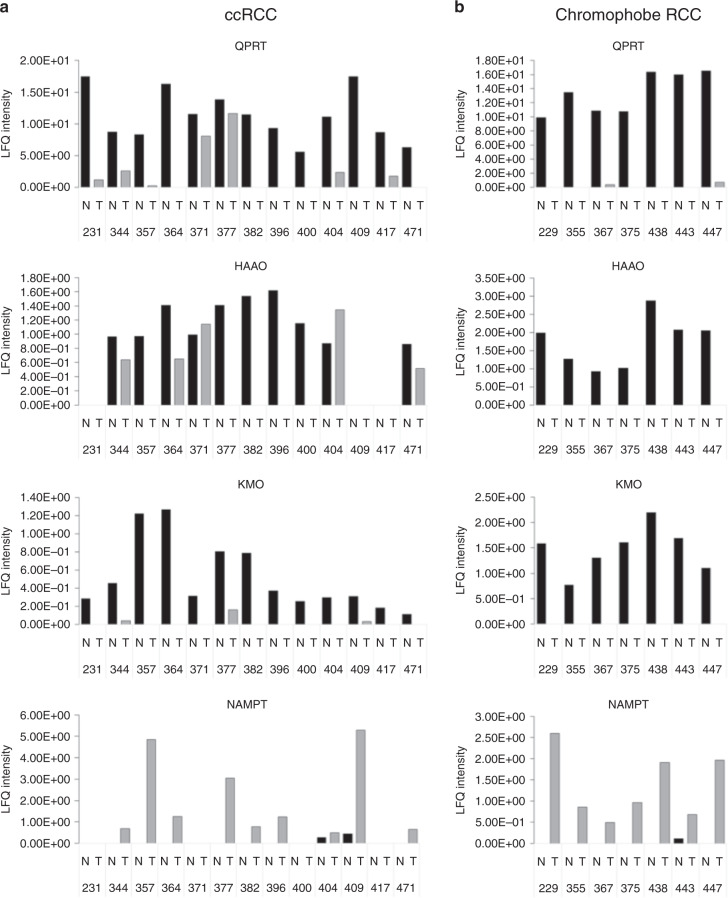
Multiple proteins in the kynurenine pathway are dysregulated in RCC. Mass spectrometric relative concentrations (LFQ intensity) of proteins in the kynurenine pathway for paired normal kidney/tumour tissue samples (black and grey bars, respectively) for **a** ccRCC and **b** chromophobe RCC. Numbers along *x*-axis refer to assigned tissue sample number. Differences between groups by Wilcoxon matched-pairs sign rank test: Clear cell RCC: QPRT (*p* < 0.001), KMO (*p* < 0.001), HAAO (*p* = 0.01), and NAMPT (*p* < 0.01); Chromophobe RCC: QPRT (*p* < 0.016), KMO (*p* < 0.016), HAAO (*p* < 0.016), and NAMPT (*p* < 0.016).

Interrogation of existing LC-MS/MS proteomic data sets across other proteins within the kynurenine pathway confirmed the decrease in QPRT in ccRCC, identifying a total of 6 unique peptides (Supplementary Table 3), which together represent 18.5% of the entire protein sequence. A consistent and significant decrease in the number of QPRT peptides identified, and in normalised (LFQ) peptide intensities (representing relative quantification), was seen in ccRCC tissues compared with normal kidney tissues, and similar findings were observed for other enzymes of the kynurenine pathway, namely 3-hydroxyanthranilite 3,4-dioxygenase (HAAO) and kynurenine 3-monoxygenase (KMO) (Fig. 3a). Conversely, nicotinamide phosphoribosyl transferase (NAMPT; which is a key enzyme in the production of NAD+ via the alternative salvage pathway) was upregulated in most tumour samples (Fig. 3a). IDO was not detected in any samples. In the data set for the seven matched pairs of chromophobe RCC versus normal kidney, very similar results as for ccRCC were obtained, with expression of QPRT, KMO and HAAO being below the level of detection in tumours (with one exception). NAMPT again showed increased expression in the tumours and was undetectable in all but one of the normal kidney tissue samples (Fig. 3b).

Four other kynurenine pathway proteins were also detected in our proteomic data sets. Kynurenine formamidase (AFMID), kynureninase (KYNU) and kynurenine aminotransferase 1 and 3 (KYAT1, KYAT3) were all observed in the chromophobe data set, while AFMID and KYAT3 were also detected in the ccRCC data. All four proteins showed a pattern of presence in normal tissue and loss in tumour tissue. However, the number of peptides detected in each sample was low (1–3), and the intensities were near to the limit of detection. For this reason, while these data suggest a pattern of altered expression for these four proteins, it should not yet be considered as conclusive.

**Fig. 4:**
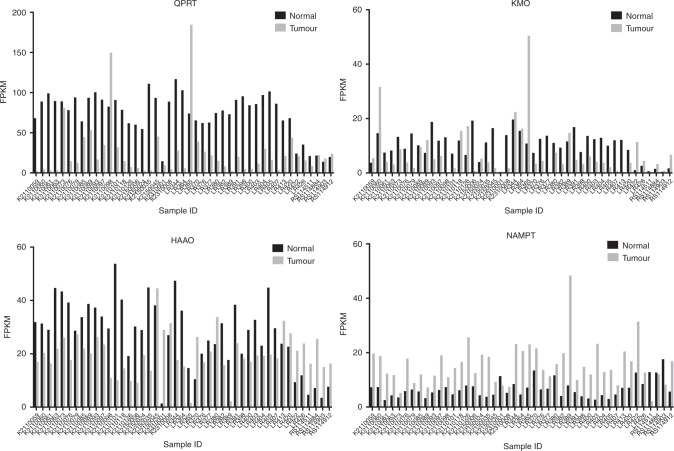
Gene expression of QPRT, KMO, HAAO and NAMPT is similarly dysregulated in clear cell RCC. Data derived from transcriptomic (RNA-seq) analysis of 45 paired normal kidney/tumour tissue samples.^30^ Numbers along *x*-axis refer to assigned tissue sample number. FPKM fragments per kilo bases of exons per million mapped reads. Differences between groups by Wilcoxon matched-pairs sign rank test: QPRT (*p* < 0.001), KMO (*p* = 0.004), HAAO (*p* = 0.001), and NAMPT (*p* < 0.001).

In addition, dysregulation of expression of QPRT, KMO, HAAO and NAMPT, largely mirroring changes observed at the protein level, was confirmed transcriptomically through examination of our previously generated RNA-seq data among 45 matched tumour (ccRCC)/normal tissue pairs (Fig. 4).^30^ Equivalent data for papillary and chromophobe RCCs are shown in Supplementary Figs, 1 and 2, respectively, based on data from The Cancer Genome Atlas Research Network.^31^

**Fig. 5:**
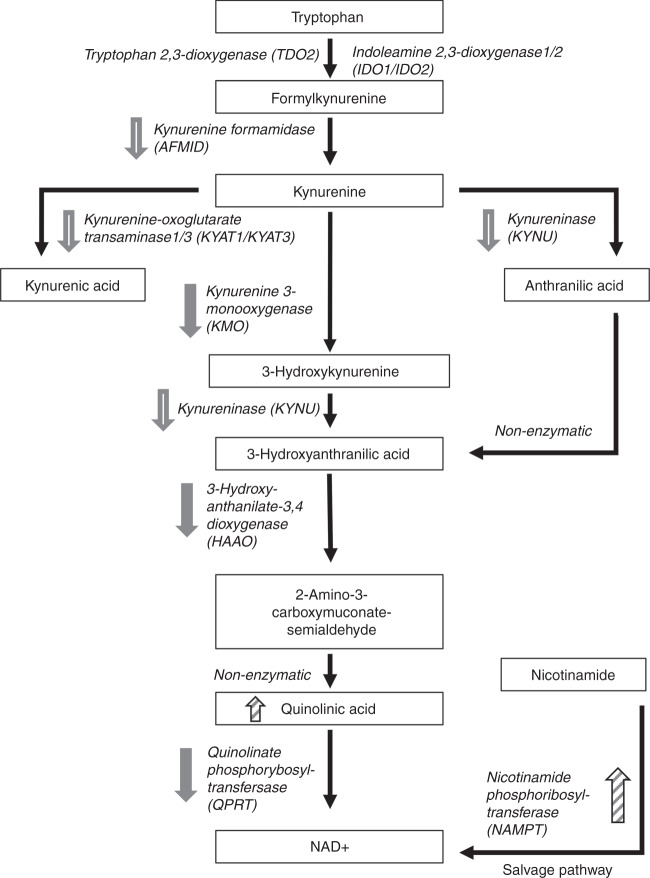
Alterations to the kynurenine pathway in RCC. The various enzymes and intermediates generated through this de novo synthesis of NAD+ from tryptophan are shown together with the salvage pathway route for generation of NAD+ from nicotinamide, catalysed by NAMPT. Enzymes for which we have strong evidence of downregulation in RCC tissues are marked with a solid grey arrow. Enzymes or substrates observed to be upregulated are marked with a hatched arrow. Enzymes with evidence to suggest they may be down regulated are marked by an open grey arrow.

The observed changes and how these impact on the kynurenine pathway are summarised in Fig. 5.

### IHC TMA analysis of the kynurenine pathway by TMA

**Fig. 6:**
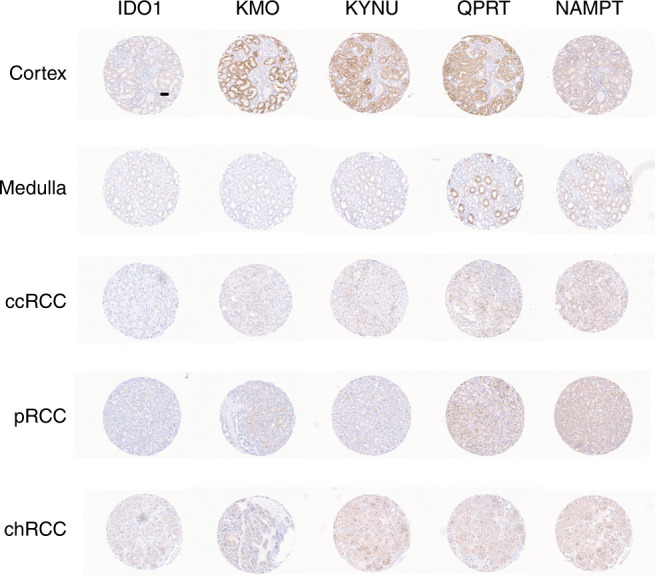
Immunohistochemical analysis of kynurenine pathway enzymes. Examples of the immunohistochemical staining patterns observed for IDO1, KMO, KYNU, QPRT and NAMPT in the normal renal cortex and medulla and different subtypes of RCC (clear cell, papillary and chromophobe) using a TMA. Scale bar (displayed in first core) = 60 μm.

In normal renal cortex, weak-to-moderate granular staining of proximal tubules (predominantly cytoplasmic) was observed for IDO1 and moderate/strong expression of KYNU, QPRT and KMO, with absent-to-moderate staining for NAMPT. In the case of KMO, staining was noticeably localised to basolateral aspect of the tubules. Glomerular reactivity was also seen for NAMPT and KYNU. In the medulla, tubules were largely negative, and two cases showed weak expression of IDO1, NAMPT and QPRT, although one of these showed distinct populations of tubules with moderate QPRT staining (Fig. 6).

In ccRCC cases, expression of IDO1, KYNU, QPRT and KMO was greatly reduced compared with normal kidney cortex and, in some cases, was absent (Fig. 6, Supplementary Table 4). IDO1 was not detected in tumour cells but only in occasional inflammatory cells or in endothelial cells, and KMO1 was also seen in some endothelial cells. For QPRT, five cases were completely negative, four cases showed only focal positivity and two weak-to-moderate staining. Conversely, NAMPT expression was absent from clear cell tumour cells in two cases but positive in five (from weak to strong) with focal positivity seen in a further four cases. Positive staining was also seen with occasional inflammatory cells and endothelial cells. Of note, the strongest staining for any of the enzymes amongst the ccRCC cases was observed in the rhabdoid cells contained in tumour 4728. A similar pattern of generally reduced staining compared with normal kidney was also seen with papillary and chromophobe cases and generally weak-to-moderate NAMPT. The findings were consistent with our MS results and demonstrate that disruption of the kynurenine pathway is a feature of RCC across histological subtypes.

## Discussion

This study provides the first comprehensive demonstration of a wide and coordinate dysregulation of the kynurenine pathway in RCC and that this is a common, unifying event, highlighting its importance in the pathogenesis of these cancers and potentially providing insights of relevance to therapeutic targeting.

The kynurenine pathway is the main route for degradation of the essential amino acid tryptophan and de novo synthesis of NAD+, generating numerous other active intermediate metabolites including kynurenine, kynurenic acid, anthranilic acid, picolinic acid and QUIN. Dysregulation of the pathway was initially highlighted in neuropsychiatric disorders but has now been implicated more widely^10^ and increasingly in tumorigenesis and immune evasion. Although in the liver, where the majority of tryptophan degradation occurs, constitutively expressed TDO2 is the initial rate-limiting enzyme in the pathway, in most other tissues IDO1 is the first and rate-limiting enzyme. Inducible by inflammatory cytokines, IDO/IDO1 has been reported to be expressed in many cancers and hypothesised to play a role in tryptophan degradation and accumulation of active metabolites in the kynurenine pathway, both of which result in T cell/immune suppression leading to the idea of IDO1 inhibitors as useful anticancer therapeutics to overcome immune resistance, for example in combination with vaccine strategies.^13,32^ However, IDO1 expression is highly dependent on tumour type, and many cancers, including renal, melanoma and thyroid, have absent or low expression of IDO1 in tumour cells in most cases.^32^ This has been confirmed subsequently with the demonstration of IDO1 expression in endothelial cells and macrophages in RCC tissues^33,34^ and a recent extensive study across many cancers where, although 80% of renal carcinomas were positive, IDO1 was absent from tumour cells and present predominantly in endothelial cells or in some lymphocyte-rich stroma.^35^ This in agreement with our IHC results and with the relatively low expression being undetectable by MS.

We initially observed that in a *VHL*+/− cell line pair, expression of QPRT increased following the introduction of *VHL*. QPRT is the final enzyme in the kynurenine pathway, converting QUIN to NAD+. However, it is apparent that this is not mainly *VHL* dependent and may be an indirect effect, since this was not seen in other VHL-transfectant cell line models and changes in QPRT expression were subsequently shown to occur in ccRCC independent of *VHL* mutation status and in chromophobe and papillary RCC tissues where *VHL* is not involved. We subsequently learnt that the 786-pRC cell line we employed also contains a *p53* mutation (personal communication from WG Kaelin to R Craven), although we do not believe this to be implicated either, since *p53* mutations are rarely seen in RCC.^3^ The underlying biology leading to such dysregulation, and whether common or divergent mechanisms are responsible, remains uncertain, but its consistency across more than one type of RCC suggests that it is a key and potentially early event.

As predicted, we found that that loss of QPRT was associated with increased QUIN in tumour tissue, and metabolomic studies of urine and tissue samples from RCC patients reported higher concentrations of quinolinate compared with healthy controls^36^ or normal tissue.^37^ QUIN has been reported to activate β-Catenin and increase proliferation in colon cancer cell lines,^38^ and in RCC cell lines, variable effects of quinolinate on cell viability or proliferation have been reported although very different quinolinate concentrations have been used across studies.^36,39^ We did not observe increased cell proliferation in our in vitro QPRT knockdown model. However, we did see a pronounced increase in anchorage-independent growth in response to loss of QPRT. It is possible that this is mediated by QUIN accumulation and exerting this effect through a potential autocrine loop involving *N*-methyl-d-aspartate receptors (NMDARs), since this receptor–ligand binding is important for its role in neurological disease^40^ and expression of NMDAR subunits has been demonstrated in the normal kidney cortex and medulla and across multiple cancer types, with receptor blockade reducing cancer cell proliferation and invasiveness in numerous cancers in vitro.^41^ QUIN is also known to be an immune modulator. For example, treatment with QUIN induced the selective apoptosis in vitro of murine thymocytes and of T helper type 1 (Th1) but not of Th2 cells, and mice treated with QUIN had significantly reduced levels of immature thymocytes in the thymus.^42^ In a microenvironment deficient in tryptophan, QUIN was found to inhibit proliferation of both lymphocytes and natural killer (NK) cells.^43^ Interestingly, accumulation of QUIN has been reported to occur in human gliomas but accompanied by increased QPRT expression and supporting NAD generation through this pathway rather than the NAMPT-mediated pathway.^44^ QPRT was shown to be induced by oxidative stress, temozolomide and irradiation and to be associated with poorer prognosis in recurrent tumours after radiochemotherapy, potentially through increasing resistance. These results suggest that targeting QPRT itself may be a potential therapeutic option and indeed data from a cell line model have implicated upregulation of QPRT as conferring resistance to NAMPT inhibitors.^45^

Examination of a parallel existing LC-MS/MS proteomic data set generated by our group as part of an ongoing proteogenomic study in RCC not only confirmed loss of QPRT in RCC but also showed loss of five other enzymes in the kynurenine pathway, namely HAAO, KMO, kynurenine formamidase and KYAT1 and 2. Remarkably little is known about these enzymes in cancer although upregulation of KMO has been described in hepatocellular carcinoma.^46^ This may be due at least in part to the lack of availability of good antibodies, at least until recently, which is why we generated our own QPRT antibody. Critically, one can expect that loss of KMO, alongside an increase in IDO expression, will lead to the accumulation in tumour tissue of kynurenine. In metabolomic studies of mouse RCC xenografts and human RCC tumours, significantly lower tryptophan and higher kynurenine levels and higher quinolinate and kynurenine levels, respectively, were seen in tumours compared with controls.^37,47^ A metabolomic study involving ccRCC along with chromophobe and papillary tissue samples also identified elevated kynurenine compared with controls but in ccRCC cases only.^48^ The immune-suppressive properties of kynurenine are well described, and it has been shown to inhibit T cell and NK cell proliferation and promote immune suppression via the aryl hydrocarbon receptor.^32,43,49^ Furthermore, it can promote cancer cell survival and motility.^38,49^ 3-Hydroxyanthranilic acid, the substrate for HAAO, has also been shown to have multiple roles in promoting tumour immune evasion, by promoting apoptosis of Th1 and NK cells, promoting differentiation of regulatory T cells and inhibiting T cell proliferation.^10,42^

This novel observation, that multiple enzymes in the kynurenine pathway downstream of IDO are all downregulated and in a manner likely to promote tumorigenesis and immune evasion, is striking. The loss of QPRT, and hence the blockade of de novo biosynthesis of NAD+ may initially seem at odds with the requirement of cells, and especially cancer cells, for this molecule.^50^ However, NAD+ may also be made from nicotinamide via the salvage pathway of which the enzyme NAMPT is a key component, and this pathway is often preferred in cancer cells.^51^ Consistent with this, we observed upregulation of NAMPT occurring as a common event in RCC, confirming a recent IHC study.^52^ As such, NAMPT forms an attractive therapeutic target, and it is of note that KPT-9274, a NAMPT/PAK4 inhibitor, has recently been reported to have activity against renal cancer xenografts.^53^

The kynurenine pathway forms a particularly attractive target for therapy, since it seems to regulate tolerance to non-self-antigens, rather than to self-antigens,^11,12^ thus reducing the risk of immune-related adverse events often seen with immune-checkpoint inhibition. Whether inhibition of IDO1 alone is sufficient to overcome pathway dysregulation is uncertain, as exemplified by the recent negative results seen with epacadostat in patients with melanoma.^14^ Indeed, a planned Phase 3 trial of this agent in combination with pembrolizumab in patients with RCC has been halted based on these findings. While combined IDO1/TDO inhibitors are currently in early Phase trials (NCT03208959), our results suggest that, in RCC at least, the kynurenine pathway is much more widely dysregulated and may require further downstream modulation in addition to IDO inhibition.

Our study has its limitations and a number of questions remain unanswered that would need to be explored in future studies. Whether the phenotypic consequences of loss or gain of QPRT extend beyond effects on colony formation, for example, and whether such changes are consistently observed across RCC cell lines, remains uncertain. Furthermore, since the 786-0 cell line is known to contain a phosphatase and tensin homologue (*PTEN*) mutation (although rarely observed in ccRCC tissues),^30^ it would be of interest to examine how deficiency of PTEN and/or dysregulation of the phosphoinositide-3 kinase/AKT/mammalian target of rapamycin signalling pathway impacts QPRT expression. We have also not examined how dysregulation of the kynurenine pathway in RCC correlates with patient outcomes.

In conclusion, we have demonstrated a widespread and apparently coordinated dysregulation of the kynurenine pathway in RCC. These findings have implications for current strategies aimed at therapeutically targeting this critical pathway and highlight the potential for novel treatment strategies, such as inhibition of NAMPT.

## Supplementary information

Supplementary Data

## Data Availability

RNA-seq data, generated through our ICGC CAGEKID study, has been deposited in a public repository as described elsewhere: Scelo et al.^30^ Proteomic data sets are available on request.
