# Supplementary material for: Dysregulation at multiple points of the kynurenine pathway is a ubiquitous feature of renal cancer: implications for tumour immune evasion
Source: Br J Cancer. 2020 May 11;123(1):137–47. doi: 10.1038/s41416-020-0874-y (PMC7341846; doi:10.1038/s41416-020-0874-y)
Supplement: Supplementary file 1 — Supplementary Data [file 41416_2020_874_MOESM1_ESM.docx]

**Supplementary Data**

Hornigold *et al*. Dysregulation at multiple points of the kynurenine pathway is a ubiquitous feature of renal cancer: implications for tumour immune evasion

**Contents**

**Supplementary Table 1.** Details of patients and tumours used in this study

**Supplementary Table 2.** Proteins identified as differentially expressed between the 786-0 +/- *VHL* cell lines

**Supplementary Table 3.** Unique peptides for the proteins in the kynurenine pathway identified by LC-MS/MS

**Supplementary Table 4.** Summary of TMA immunohistochemical staining for kynurenine pathway components

**Supplementary Figure 1.** Gene expression data for QPRT, KMO, HAAO and NAMPT in papillary RCC (TCGA data)

**Supplementary Figure 2.** Gene expression data for QPRT, KMO, HAAO and NAMPT in chromophobe RCC (TCGA data)

**Supplementary Table 1.** Details of patients and tumors used in this study. In all cases, percentage tumor necrosis was low (<10%), and the percentage of viable tumor cells was >70%, except for one sample (50%). The samples used in the TMA are not included here but details are provided in Supplementary Table 4 with the IHC results.

| **Tumor ID** | **Age** | **Sex** | **Type** | **Grade** | **TNM Stage** | **Western Blot** | **IHC for QPRT** | **LC-MS/MS** | **Quinolinic acid concentration** |
| --- | --- | --- | --- | --- | --- | --- | --- | --- | --- |
| 28 | 79 | F | Clear Cell | 2 | T3bN0M0 |  | x |  |  |
| 42 | 40 | M | Clear Cell | 3 | T3bN0M1 | x | x |  |  |
| 51 | 67 | M | Clear Cell | 3 | T3bN0M0 | x |  |  |  |
| 53 | 55 | M | Clear Cell | 3 | T3bN0M1 | x | x |  |  |
| 64 | 48 | M | Clear Cell | 2 | T1a,N0,M1 |  |  |  | x |
| 77 | 61 | M | Clear Cell | 3 | T4N0M1 | x |  |  |  |
| 79 | 57 | M | Clear Cell | 3 | T2N0M1 | x | x |  | x |
| 81 | 63 | M | Clear Cell | 2 | T3aN0M0 |  | x |  |  |
| 82 | 57 | M | Clear Cell | 3 | T1aN0M0 |  | x |  |  |
| 87 | 63 | F | Clear Cell | 3 | T1bN0M0 | x |  |  |  |
| 88 | 70 | M | Clear Cell | 4 | T2N0M0 |  | x |  |  |
| 98 | 60 | F | Clear Cell | 3 | T3bN2M1 | x | x |  |  |
| 117 | 77 | M | Clear Cell | 4 | T3aN0M0 |  | x |  | x |
| 137 | 64 | M | Clear Cell | 3 | T3bN0M0 | x |  |  |  |
| 138 | 73 | M | Clear Cell | 2 | T1aN0M0 | x |  |  |  |
| 150 | 72 | M | Clear Cell | 3 | T3aN0M0 | x |  |  |  |
| 151 | 58 | M | Clear Cell | 4 | T1bN0M0 |  | x |  |  |
| 153 | 71 | F | Clear Cell | 2 | T1bN0M0 |  | x |  |  |
| 156 | 49 | F | Clear Cell | 3 | T1bN0M0 |  | x |  |  |
| 157 | 67 | M | Clear Cell | 4 | T1aN0M1 |  | x |  |  |
| 172 | 51 | M | Clear Cell | 4 | T1bN1M1 |  |  |  | x |
| 231 | 52 | F | Clear Cell | 4 | T3aN2M1 |  |  | x |  |
| 344 | 66 | F | Clear Cell | 2 | T1aN0M0 |  |  | x |  |
| 357 | 46 | F | Clear Cell | 4 | T3aN1M1 |  |  | x |  |
| 364 | 77 | F | Clear Cell | 3 | T1aN0M0 |  |  | x |  |
| 370 | 54 | M | Clear Cell | 4 | T3bN0M0 |  |  | x |  |
| 371 | 67 | M | Clear Cell | 3 | T1bN0M0 |  |  | x |  |
| 377 | 59 | F | Clear Cell | 4 | T3aN0M0 |  |  | x |  |
| 382 | 73 | M | Clear Cell | 3 | T1bN0M0 |  |  | x |  |
| 396 | 69 | M | Clear Cell | 2 | T3aN0M0 |  |  | x |  |
| 400 | 54 | F | Clear Cell | 2 | T1aN0M0 |  |  | x |  |
| 404 | 65 | F | Clear Cell | 4 | T3aN0M0 |  |  | x |  |
| 409 | 62 | F | Clear Cell | 4 | T2aN0M0 |  |  | x |  |
| 417 | 49 | F | Clear Cell | 2 | T1aN0M0 |  |  | x |  |
| 471 | 66 | M | Clear Cell | 3 | T3aN0M0 |  |  | x |  |
| 229 | 56 | M | Chromophobe | NA | T1aN0Mo |  |  | x |  |
| 355 | 59 | M | Chromophobe | NA | T1bN0M0 |  |  | x |  |
| 367 | 81 | F | Chromophobe | NA | T1bN0M0 |  |  | x |  |
| 375 | 60 | M | Chromophobe | NA | T3aN0M0 |  |  | x |  |
| 348 | 63 | M | Chromophobe | NA | T2N0M0 |  |  | x |  |
| 443 | 52 | M | Chromophobe | NA | T2bNxMx |  |  | x |  |
| 447 | 60 | M | Chromophobe | NA | T2bN0M0 |  |  | x |  |

NA – not applicable

**Supplementary Table 2.** Proteins identified as differentially expressed between the 786-0 +/- *VHL* cell lines by 2D-PAGE and mass spectrometric sequencing of excised spots.

| **Spot ID** | **Protein name** | **Accession No.** | **Theoretical**  **values** |  | **Observed**  **values** |  | **Mean fold change**  **+VHL / -VHL**  **(n=3)** |
| --- | --- | --- | --- | --- | --- | --- | --- |
|  |  |  | **MW (kDa)** | **pI** | **MW (kDa)** | **pI** |  |
|  |  |  |  |  |  |  |  |
| W1 | Quinolinate phosphorylribosyltransferase | Q15274 | 30.8 | 5.8 | 36.1 | 6.0 | Present / absent |
| W6 | Heterogeneous nuclear ribonucleoprotein F | P52597 | 45.7 | 5.38 | 51.9 | 5.5 | 2.8 ↑ |
| W7 | 78 kDa glucose-regulated protein | P11021 | 70.5 | 5.01 | 76.0 | 5.1 | 2.7 ↑ |
| W8 | 94 kDa glucose-regulated protein | P14625 | 90.2 | 4.73 | 58.3 | 4.8 | 2.1↑ |
| W9 | EIF-5A (translation initiation factor) | P10159 | 16.7 | 5.08 | 18.7 | 5.2 | 1.9 ↑ |
| W10 | Thioredoxin peroxidase |  |  |  | 26.7 | 5.7 | 1.7 ↑ |
| P1 | Keratin 19 | P08727 | 44.1 | 5.05 | 45.9 | 5.1 | 33.3 ↓ |
| P3 | EIF 4A | P60842 | 46.2 | 5.32 | 51.7 | 5.5 | 6.6 ↓ |
| P4 | Calumenin | O43852 | 35.0 | 4.46 | 50.2 | 4.4 | 4.6 ↓ |
| P5/P10 | Transaldolase 1 | P37837 | 37.5 | 6.36 | 39.0/37.3 | 6.6/6.1 | 3.8 ↓ / 1.7 ↓ |
| P8 | Glutamate carboxypeptidase | Q96KP4 | 52.9 | 5.66 | 56.6 | 6.0 | 2.1 ↓ |
| P11 | Heat shock protein 27 kDa | P04792 | 22.8 | 5.98 | 30.4 | 5.8 | 1.6 ↓ |
| P12 | ATP synthase beta chain mitochondrial F1 complex | P06576 | 51.8 | 5.00 | 54.5 | 5.0 | 1.5 ↓ |
| P13 | 78 kDa glucose-regulated protein (BiP) | P11021 | 70.5 | 5.01 | 75.0 | 5.1 | 1.6 ↓ |
| P15 | Myosin light chain 3 | P08590 | 21.8 | 5.03 | 15.9 | 4.4 | 5.1↓ |
| P17 | Cathepsin D | P07339 | 37.9 | 5.60 | 32.0 | 5.2 | 2.7 ↓ |
| P22 | Ubiquinol cytochrome c reductase core protein 1 | P31930 | 49.1 | 5.43 | 51.7 | 5.7 | 4.0 ↓ |
| P23 | Swiprosin 1 | Q96C19 | 26.7 | 5.15 | 32.2 | 5.2 | 2.7 ↓ |
| P29 | Vesicle amine transport protein 1 | Q99536 | 32.6 | 6.45 | 51.2 | 6.2 | 1.5 ↓ |

**Supplementary Table 3.** Unique peptides for the proteins in the kynurenine pathway identified by LC-MS/MS in the two proteomic studies for ccRCC and normal kidney tissue and for chromophobe RCC and normal kidney

| **Clear cell RCC** | |
| --- | --- |
| **Gene Name/PROTEIN** | **PEPTIDES** |
| QPRT | DNHVVAAGGVEK, GAGWTGHVAGTR, GAGWTGHVAGTRK, VALNTLAR, YDLGGLVMVK, YGLLVGGAASHR |
| HAAO | DLGTQLAPIIQEFFSSEQYR, EPPFPLSTR, FANTVGLVVER, FANTVGLVVERR, LMHQEQLK, QGEIFLLPAR, RLETELDGLR, SIMEPMSLDAWLDSHHR, TGKPIPDQLLK, TGKPIPDQLLKEPPFPLSTR |
| KMO | AHVNSSWFIFQK, AVDSLEQISNLISR, AVGLEDQIVSQGIPMR, DLLTAAEKYPNVK, ENLNKDLLTAAEKYPNVK, LLTSNDVVDFFQK, MDSSVIQR, NFQIDVYEAR, NGDYAMEPNYLHIWPR, NTFMMIALPNMNK, SINLALSHR, SQYILSVSR, |
| NAMPT | AVPEGFVIPR, DPVADPNKR, DVYKEHFQDDVFNEK, GTDTVAGLALIK, GTDTVAGLALIKK, GVSSQETAGIGASAHLVNFK, KFPVTENSK, MWSIENIAFGSGGGLLQK, NAQLNIELEAAHH, STQAPLIIRPDSGNPLDTVLK, SYSFDEIRK, TPAGNFVTLEEGKGDLEEYGQDLLHTVFK, VIQGDGVDINTLQEIVEGMK, VLEILGK, YDGHLPIEIK, YLLETSGNLDGLEYK, YLLETSGNLDGLEYKLHDFGYR |
| AFMID | LGAEEALR, VLVVVGQFDSPEFHR |
| KYAT1 | Not detected |
| KYAT3 | IAAIDSLNQYTR, LAADPSVVNLGQGFPDISPPTYVKEELSK, MAGATPVFIPLR, WSSSDWTLDPQELESK |
| TDO | Not detected |
| IDO | Not detected |
| KYNU | Not detected |
| **Chromophobe RCC** | |
| QPRT | CSGIASAAAAAVEAAR, DNHVVAAGGVEK, EDCPGLNYAALVSGAGPSQAALWAK, GAGWTGHVAGTR, GPAHCLLLGER, MDAEGLALLLPPVTLAALVDSWLR, VALNTLAR, YDLGGLVMVK, YGLLVGGAASHR |
| HAAO | DLGTQLAPIIQEFFSSEQYR, EPPFPLSTR, FANTVGLVVER, GSFQPPVCNK, LMHQEQLK, QGEIFLLPAR, RLSLAPDDSLLVLAGTSYAWER, RRLETELDGLR, SIMEPMSLDAWLDSHHR, TGKPIPDQLLK, TQGSVALSVTQDPACK, VMFIGGPNTR |
| KMO | AHVNSSWFIFQK, AVDSLEQISNLISR, AVGLEDQIVSQGIPMR, DLLTAAEKYPNVK, ENLNKDLLTAAEKYPNVK, FSNDLSLCLPVFSR, IRYHEAVQR, LLTSNDVVDFFQK, MDSSVIQR, NGDYAMEPNYLHIWPR, NTTCFPAK, RNFQIDVYEAR, RPWNWIAHFR, SINLALSHR, SQYILSVSR, YFPDAIPLIGEK, YHEAVQR |
| NAMPT | DLLNCSFK, DPVADPNKR, DVYKEHFQDDVFNEKGWNYILEKYDGHLPIEIK, FPVTENSKGYK, GTDTVAGLALIK, GTDTVAGLALIKK, KFPVTENSK, KFPVTENSKGYK, LHDFGYR, MWSIENIAFGSGGGLLQK, STQAPLIIRPDSGNPLDTVLK, SYSFDEIRK, TPAGNFVTLEEGKGDLEEYGQDLLHTVFK, VIQGDGVDINTLQEIVEGMK,  VIQGDGVDINTLQEIVEGMKQK, VKYEETVFYGLQYILNK, VLEILGK,  VYSYFECR, YDGHLPIEIK, YLLETSGNLDGLEYK,  YLLETSGNLDGLEYKLHDFGYR |
| AFMID | DNVLTQIILK, LGAEEALR, MSAEELENQYCPSR, TYSQIGIEATTR, VAQAQPVDPTCR, VLVVVGQFDSPEFHR |
| KYAT1 | ALVLNTPNNPLGK |
| KYAT3 | AIILNTPHNPLGK, IAAIDSLNQYTR, LAADPSVVNLGQGFPDISPPTYVKEELSK, LGWSIGPNHLIK, LSAIPVSAFCNSETK, MAGATPVFIPLR |
| TDO | Not detected |
| IDO | Not detected |
| KYNU | IAAYGHEVGK, KPVVNIITPSHVEER, LQLIPGVCGFR, MEPSSLELPADTVQR, VALHLDEEDKLR, VAPVPLYNSFHDVYK, YLNAGAGGIAGAFIHEK |

**Supplementary Table 4.** Summary of TMA immunohistochemical staining for kynurenine pathway components. Results are for tumor cell positivity with occasional inflammatory cell and blood vessels staining as described in the texted.

|  | **12437** | **8464** | **16780** | **7029** | **19467** | **451** | **13216** | **5141** | **4728** | **18738** | **11513** |  | | **67** | | **2731** | **7314** | **19636** | **19813** | **17276** |  | **14949** | **18299** | **25823** |
| --- | --- | --- | --- | --- | --- | --- | --- | --- | --- | --- | --- | --- | --- | --- | --- | --- | --- | --- | --- | --- | --- | --- | --- | --- |
| **Subtype** | Clear cell (conventional) | | | | | | | | | | | |  | | Papillary | | | | | |  | Chromophobe | | |
| **Grade & Stage** | G3 stage I |  | G4 stage I | G3 stage III | G4 stage IV | G4 stage I | G4 stage IV | G4 stage IV (sarc) | G4 stage III (rhab) | G2 stage I | G2 stage I |  | | Type 1 G2 stage I | | G3 stage I | Type 1 G2 stage I | Type 2 G2 stage II | Type 2 stage I | G2 stage I |  | stage I | stage I | stage IV |
|  |  |  |  |  |  |  |  |  |  |  |  |  | |  | |  |  |  |  |  |  |  |  |  |
| **IDO1** | - | - | - | - | - | - | - | - | - | - | - |  | | - | | - | - | - | - | - |  | - | - | - |
| **KMO** | - | - | +/++ | - | +/++ | - | -/++ (focal ~50%) | - | -/++ (focal <50%) | - | -/+ (focal ~30%) |  | | - | | - | -/+ (focal) | -/+ (focal) | - | - |  | - | +/++ | -/+ (focal ~10%) |
| **KYNU** | - | - | - | +/++ | + | - | +/++ | -/+ | +/++ | -/+ | -/+ (<10%) |  | | + | | - | - | + | - | -/+ |  | -/+ (focal) | -/++ (focal) | +/++ |
| **QPRT** | - | - | - | - | -/+ (focal <50%) | -/+ (focal <50%) | - | +/++ | -/++ (focal <50%) | -/++ (focal >50%) | +/++ |  | | -/+ (focal) | | - | -/++ (focal) | +/++ | - | - |  | -/+ (focal <10%) | -/+ (focal 20%) | -/+ |
| **NAMPT** | - | - | + | -/+ (focal) | +/++ | + | +/++ | -/+ (focal) | ++/+++ | -/+/++ (focal >70%) | -/+ |  | | + | | + | + | +/++ | -/+ | +/++ |  | -/+ | -/+ | + |

(sarc) = sarcomatoid elements noted

(rhab) = rhabdoid elements noted

**Supplementary Figure 1. Gene expression data for QPRT, KMO, HAAO and NAMPT in papillary RCC**. Based on data from the TCGA Research Network of 30 paired normal kidney/tumor tissue samples (black and grey bars, respectively). Numbers along x-axis refer to assigned tissue sample number. FPKM - fragments per kilo bases of exons per million mapped reads. Differences between groups by Wilcoxon matched-pairs sign rank test: QPRT (p=0.76), KMO (p<0.001), HAAO (p<0.001) and NAMPT (p<0.001)

**
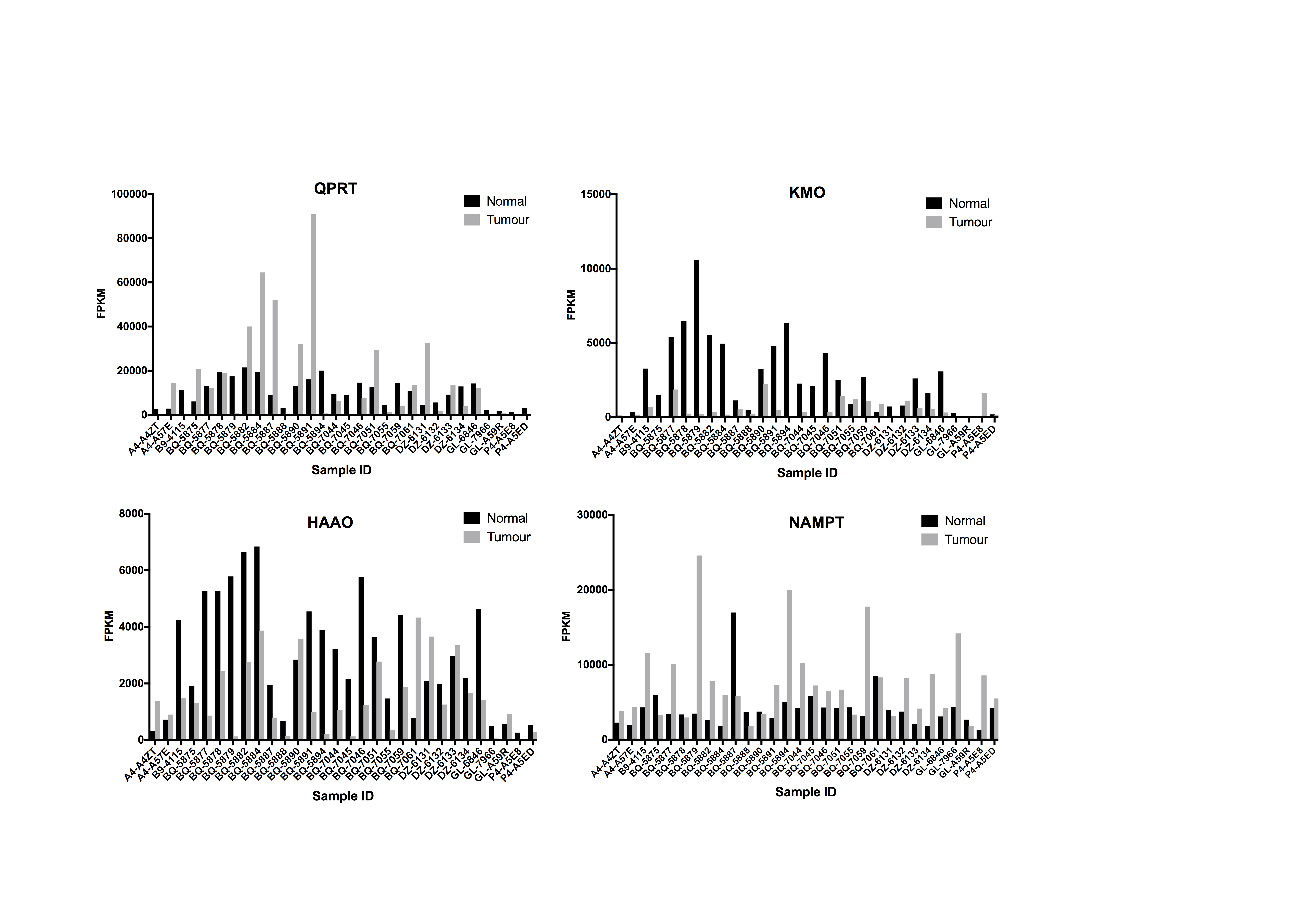
**

**Supplementary Figure 2. Gene expression data for QPRT, KMO, HAAO and NAMPT in chromophobe RCC**. Based on data from the TCGA Research Network of 25 paired normal kidney/tumor tissue samples (black and grey bars, respectively). Numbers along x-axis refer to assigned tissue sample number. FPKM - fragments per kilo bases of exons per million mapped reads. Differences between groups by Wilcoxon matched-pairs sign rank test: QPRT (p<0.001), KMO (p<0.001), HAAO (p<0.001) and NAMPT (p=0.69).

**
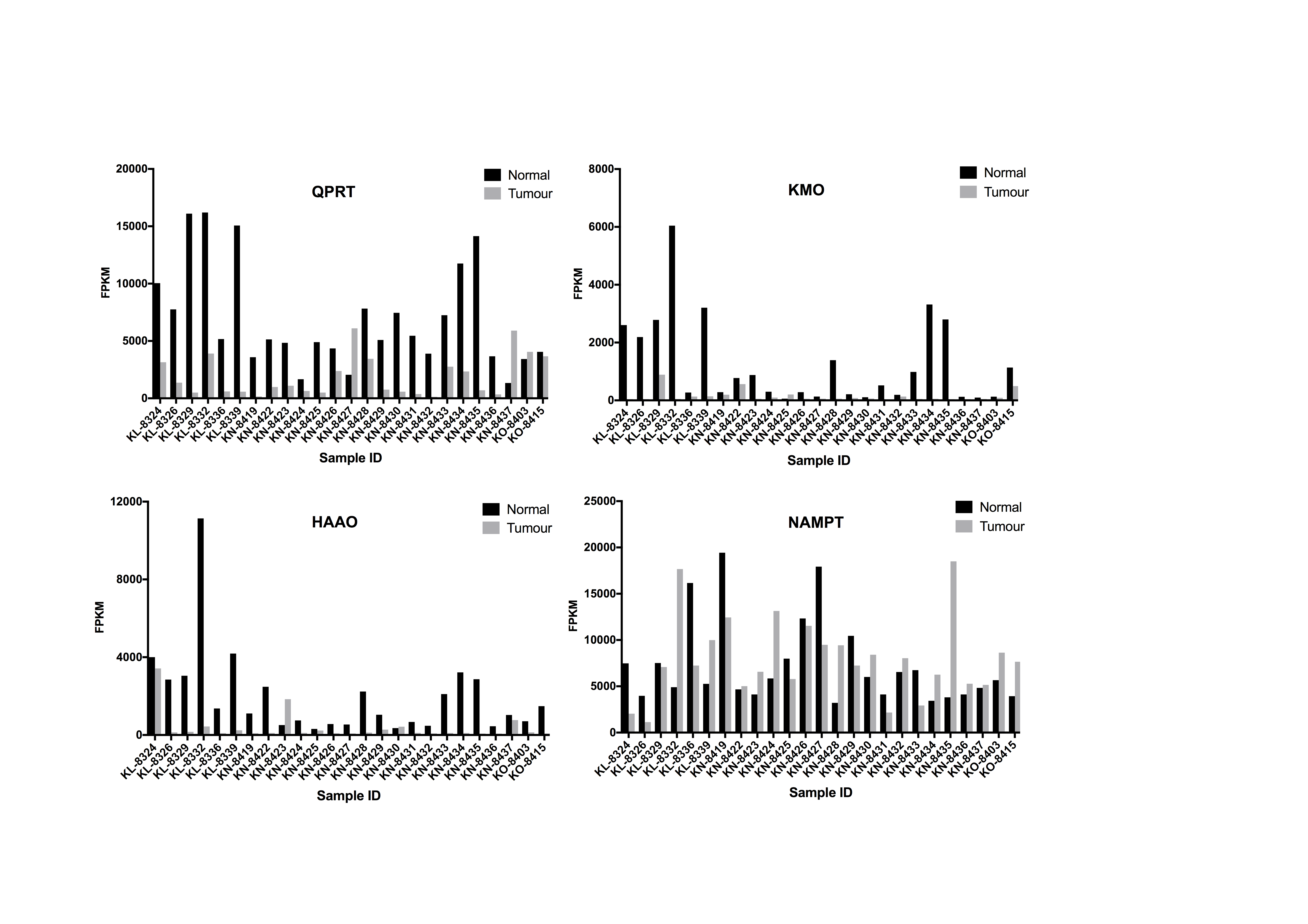
**
